# Nanoscale mapping of optically inaccessible bound-states-in-the-continuum

**DOI:** 10.1038/s41377-021-00707-2

**Published:** 2022-01-20

**Authors:** Zhaogang Dong, Zackaria Mahfoud, Ramón Paniagua-Domínguez, Hongtao Wang, Antonio I. Fernández-Domínguez, Sergey Gorelik, Son Tung Ha, Febiana Tjiptoharsono, Arseniy I. Kuznetsov, Michel Bosman, Joel K. W. Yang

**Affiliations:** 1 Institute of Materials Research and Engineering, A*STAR (Agency for Science, Technology and Research), 2 Fusionopolis Way, #08-03 Innovis, 138634 Singapore, Singapore; 2 Department of Materials Science and Engineering, National University of Singapore, 9 Engineering Drive 1, 117575 Singapore, Singapore; 3 Singapore University of Technology and Design, 8 Somapah Road, 487372 Singapore, Singapore; 4 Departamento de Física Teórica de la Materia Condensada and Condensed Matter Physics Center, Universidad Autónoma de Madrid, 28049 Madrid, Spain; 5 Singapore Institute of Food and Biotechnology Innovation, A*STAR (Agency for Science, Technology and Research), 31 Biopolis Way, #01-02 Nanos, 138669 Singapore, Singapore

**Keywords:** Nanophotonics and plasmonics, Nanocavities

## Abstract

Bound-states-in-the-continuum (BIC) is an emerging concept in nanophotonics with potential impact in applications, such as hyperspectral imaging, mirror-less lasing, and nonlinear harmonic generation. As true BIC modes are non-radiative, they cannot be excited by using propagating light to investigate their optical characteristics. In this paper, for the 1st time, we map out the strong near-field localization of the true BIC resonance on arrays of silicon nanoantennas, via electron energy loss spectroscopy with a sub-1-nm electron beam. By systematically breaking the designed antenna symmetry, emissive quasi-BIC resonances become visible. This gives a unique experimental tool to determine the coherent interaction length, which we show to require at least six neighboring antenna elements. More importantly, we demonstrate that quasi-BIC resonances are able to enhance localized light emission via the Purcell effect by at least 60 times, as compared to unpatterned silicon. This work is expected to enable practical applications of designed, ultra-compact BIC antennas such as for the controlled, localized excitation of quantum emitters.

## Abstract (web summary)

The optically inaccessible bound-states-in-the-continuum (BIC) is probed via sub-1-nm electron beam, where the cathodoluminescence emissions from Si nanoantenna arrays are able to reveal the coherent interaction length of quasi-BIC resonances.

## Graphical abstract

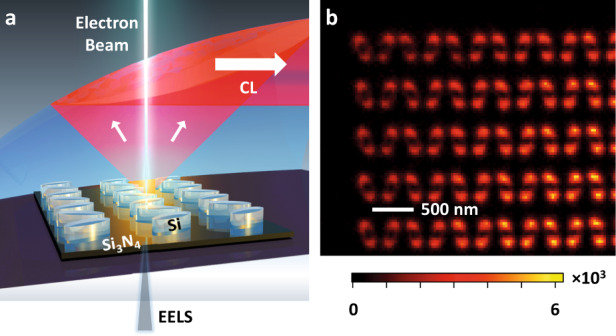

## Introduction

Bound-states-in-the-continuum (BIC) is a fascinating concept that has its origins in quantum mechanics in 1929^1^. Counterintuitively, optical modes with energies higher than the potential wells, i.e., in the freely propagating continuum, can still be localized and bound in space when these wells are appropriately designed^1^. This BIC concept was first demonstrated experimentally in semiconductor heterostructures in 1992^2^, based on earlier theoretical predictions^3,4^. To date, the concept of BIC has been generalized as a wave phenomenon into various fields, such as acoustics^5,6^, microwave physics^7,8^, and optics^9–13^. BIC in optics gives remarkably narrow resonances that are controlled by the design of nanostructures, such as dielectric gratings^9^, arrays of coupled waveguides^10,11^, layered nanoparticles^12^, and photonic crystal slabs^13^. The general principle of BIC is the complete suppression of radiative losses in the far-field by total destructive interference of radiation from the charge-current configuration associated with the mode^14,15^. These BIC modes have energies above the light line, hence the “continuum” denomination, but are completely “dark” in the sense that they do not couple to radiative modes in free-space but are instead bound to the structures that support them.

Recently, BIC has been explored in nanophotonics mostly with nanoantenna arrays^16–22^, but in individual resonators as well^12,23–25^. Nanoantenna designs with broken symmetry provide small but sufficient coupling to form *quasi-BIC* modes that enable far-field optical excitation, while *true BIC* modes do not couple to the far-field^17–22,26^. As the (quasi-) BIC modes are supported by nanoantennas, controlling their geometry can achieve unidirectional scattering^27^, sensitive hyperspectral imaging^21^, lasing^28–30^, nonlinear nanophotonics^20,24,31,32^, chirality^33^, bio-sensing^21,22,34^, and topological photonics^26,27^. However, due to the “dark” nature of the true BIC resonances, optical excitation in the far-field is unable to reveal its mode characteristics. In addition, although it has been demonstrated that a finite antenna array with only 8 × 8 elements is able to achieve BIC lasing under far-field optical excitation^35^, no direct characterization technique has determined the characteristic length that is required to setup a BIC mode, or rather how quickly the BIC mode decays as it approaches the edge of a large array.

BIC modes have no coupling to free-space radiation and are therefore optically inaccessible, except for the quasi-BIC modes. Experiments that attempt to measure BIC modes will therefore need to be sensitive to both the near-field and the far-field simultaneously; in this way, the “true”, non-radiative BIC modes can be distinguished from the quasi, radiative BIC modes. To this aim, our experiments combine cathodoluminescence (CL) and monochromated electron energy loss spectroscopy (EELS) in a scanning transmission electron microscope (STEM). The nanometer-sized, focused electron beam is used to excite the optical modes in the near-field. The energy transferred in these radiative and non-radiative excitations is measured with EELS, while only radiative losses are measured with CL in the far-field. A comparison of both EELS and CL spectra distinguishes the lossy and the trapped optical modes, providing an experimental setup to unambiguously characterize true photonic BICs with nanometer spatial precision.

In this work, we provide experimental and theoretical evidence of true BIC resonances that are locally excited by a nanometer electron beam in the STEM and directly probed using EELS and CL spectroscopy. Localized plasmonic optical transitions have been investigated before in the STEM^36–39^. The electron microscope was shown to provide a unique combination of broadband, near-field excitation, wide-range spectroscopy, and ultra-high spatial resolution, enabling a comprehensive analysis of optical resonances. Here, we use it to probe the BIC mode on designed arrays of silicon nanoantennas. By combining the spectroscopic techniques CL and EELS in the STEM, we provide direct experimental evidence of “true” BIC modes. We place “true” in quotation marks, as our fabricated arrays are necessarily of finite size, while true BIC modes in the strict term are only fully developed in infinitely large arrays. Numerical simulations as well as multipolar decomposition analysis support our claim that quasi- and “true” BIC modes can be distinguished.

## Results

**Fig. 1:**
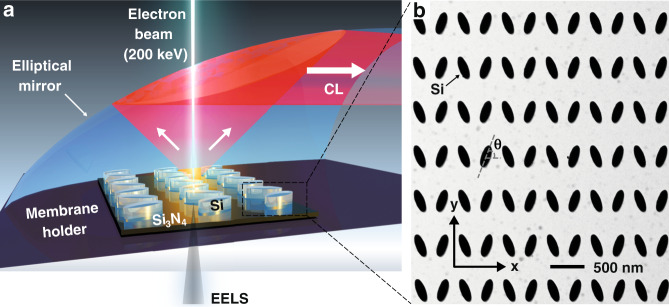
Experimental setup for exciting and probing bound-states-in-the-continuum (BIC) modes via a sub-1-nm electron beam. **a** Schematic of the experimental setup in a STEM where a high-energy electron beam focused to ~1 nm is used to probe an array of Si nanoantennas on a 30-nm thick suspended Si_3_N_4_ membrane. Complementary measurements of the energy lost by electrons, and energy of emitted photons result in EELS and CL spectra respectively. **b** Bright-field STEM image of an amorphous Si nanoantenna array supporting quasi-BIC modes on a 30-nm-thick Si_3_N_4_ membrane. “True” BIC occurs when $\theta = 90^\circ$

Figure 1a shows the experimental setup for probing the BIC mode, where a high-energy electron beam with a diameter of ~1 nm in the STEM is focused onto an array of Si nanoantennas, lithographically defined on a 30-nm-thick suspended amorphous Si_3_N_4_ membrane (see Fig. S1 and Methods section for fabrication details). When this focused electron beam is positioned near a nanostructure, a fraction of the energy of the electron beam will be used to polarize the free and bound electrons within the material that start to oscillate according to the modes supported by the nanostructures. These resonant modes can subsequently lose their energy to free-space emission, which is measured with CL^40–43^, as shown previously for metallic nanostructures^43–49^ and silicon nanoantennas^50–54^. All the losses—radiative and non-radiative—that the electron beam encounters in the excitation of the resonant modes are measured experimentally with EELS, a true near-field technique. By combining both CL and EELS spectroscopy techniques in the STEM, we will demonstrate the possibility of measuring BIC modes at the nanometer length scale. Our universal approach to distinguish all the bright and dark optical modes has the additional advantage of performing simultaneous STEM imaging with nanometer resolution, to visualize the local sample morphology. A more detailed description of the experimental setup for measuring “true” and quasi-BIC modes is given in the Methods section.

**Fig. 2:**
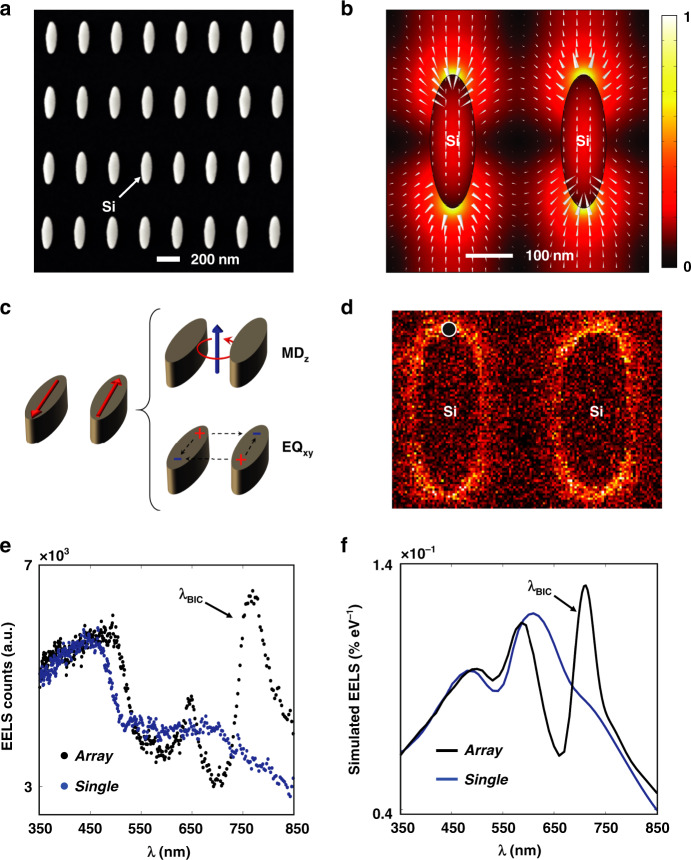
Probing of a “true” BIC resonance mode by Electron Energy Loss Spectroscopy. **a** STEM annular dark-field image of a silicon nanoantenna array with elliptic cylinders aligned at a tilt angle θ = 90°. **b** Simulated near-field mode pattern of the “true” BIC mode, with arrows indicating the electric field direction; their length being proportional to the amplitude. **c** Schematic of the multipole moments comprising the “true” BIC mode: the *z*-polarized magnetic dipole (MD_*z*_) and the electric quadrupole (EQ_*xy*_). **d** Experimental EELS map of the elliptic cylinder nanoantenna array with the “true” BIC mode resembling the E-field maps in (**b**). **e** EELS spectrum (black color) as measured at the position near the tip of the ellipse as indicated in (**d**) demonstrating the excitation of the “true” BIC mode. For benchmarking, the EELS spectrum measured from an isolated single antenna element of the same size (blue color) clearly shows the missing peak associated with the BIC mode. More information about the single antenna measurements can be found in the Fig. S3. **f** FEM-simulated EELS spectra for the nanoantenna array (black), showing a BIC resonance, and for a single antenna element (blue), showing no BIC resonance

Figure 1b shows a STEM image of an array of Si elliptic cylinder nanoantennas that support (quasi-) BIC modes. The designed dimensions for the long-axis and short-axis of the antennas are 286 nm and 96 nm, respectively, with a pitch size of 562 nm. The tilt angle θ of the nanoantennas is commonly used to control the degree of symmetric breaking of the coupled nanoantennas^19^, providing far-field optical access to the quasi-BIC mode. As θ approaches 90° (see the STEM image in Fig. 2a), the silicon nanoantenna array supports a “true” BIC mode that is not optically accessible from the far-field. The near-field pattern of this “true” BIC mode can be found by computing the eigenmodes of the system. Figure 2b shows the simulation results, corresponding to a BIC mode at the Gamma symmetry point at a wavelength of ~720 nm and a quality factor (*Q*-factor) of ~90. Note that the finite bandwidth of the mode is due to the dissipative loses (i.e., the extinction coefficient *k* = 0.01) of silicon at that wavelength; the measured *n* and *k* values for the amorphous silicon film are shown in Fig. S2. This finite *Q*-factor value of the “true” BIC mode is consistent with the ones reported in the literature^55^, in which they analyzed the limited *Q*-factor of BICs imposed by absorption in silicon. In addition, the near-field oscillation characteristic of this BIC mode is shown in the supporting video file “NF_BIC.gif”, where the neighboring unit cells are oscillating in phase when *k* = 0. The schematic in Fig. 2c shows the equivalent multipole moments that give rise to the BIC, consisting of a *z*-polarized magnetic dipole (MD_*z*_) and an electrical quadrupole (EQ_*xy*_). Due to its non-radiative nature, this “true” BIC resonance mode can neither be excited nor measured using far-field techniques^23^. In our experimental setup, the electron energy losses measured with monochromated EELS would detect this BIC mode in the near-field. On the other hand, in situ CL measurements that collect emitted light will allow us to determine if this mode can be optically detected in the far-field.

**Fig. 3:**
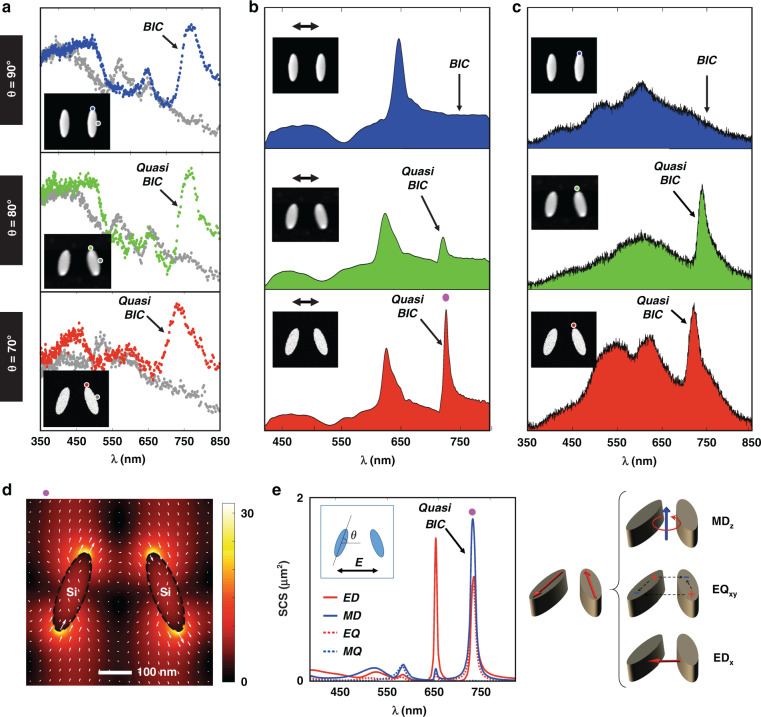
Evolution of CL and EELS spectra for nanoantenna arrays with different tilt angles. **a** Measured EELS spectra, **b** reflectance spectra under *x*-polarized incidence condition, and **c** CL spectra from the nanoantenna array with different tilt angle θ of 90°, 80°, and 70°. **d** Spatial distribution of the electric field |***E***| at the quasi-BIC resonance at ~720 nm for the nanoantenna array with a tilt angle θ of 70°. The arrows denote the electric field direction, the length of the arrows being proportional to the amplitude. **e** Multipolar decomposition of the scattering cross-section (SCS) and corresponding schematics of the multipoles excited at the quasi-BIC mode

EELS measurements of a “true” BIC mode are presented in Fig. 2. The measured EELS BIC map in Fig. 2d and EELS spectra in Fig. 2e show clear evidence of a BIC mode in the silicon antenna array, while this mode is strikingly absent when EELS is measured on individual antennas that are not part of an array, confirming the interpretation of the array effect. To corroborate these experimental observations, Fig. 2f presents finite-element-method (FEM)-based COMSOL simulations of EELS spectra for a single antenna and for antennas in an array (see Methods). The simulation verifies the observed difference between the antenna array and the single element. Moreover, the first row in Fig. 3b presents optical reflectance measurements under *x*-polarized incidence condition (see the *x*–*y* coordinate in the STEM image in Fig. 1b), where no BIC resonance feature is observed at ~720 nm in the case of a 90° tilt angle due to its non-radiative nature. Final evidence is provided in Fig. 3c where no BIC resonance is observed in the CL far-field emission, though the EELS data in Fig. 3a clearly show that the mode is excited by the electron beam. This is direct verification of the non-radiative nature of this “true” BIC mode.

In an attempt to distinguish the “true” bound states from the emissive quasi-BIC resonances, we patterned symmetry-broken arrays of the same nanoantennas by changing the tilt angle θ of the nanoantennas from 90°, to 80°, and 70°. Figure 3a presents the measured EELS spectra showing both the “true” BIC resonance at θ = 90° and the quasi-BIC resonances at θ = 80° and θ = 70°. The quasi-BIC mode now becomes visible both in the reflectance spectra and in the CL emission spectra, as shown in Fig. 3b, c, while this mode is not present for the “true” BIC at θ = 90°. For completeness, the simulated reflectance spectra, the EELS maps for the quasi-BIC mode at θ = 70°, and the substrate effects due to varying Si_3_N_4_ membrane thicknesses are shown in Figs. S4-6, respectively. In addition, Fig. S7 presents angle-resolved reflectance spectra under *x*-polarization and it shows that the true BIC modes for the elliptic cylinder nanoantenna pairs with a tilt angle θ of 90° are inaccessible by far-field propagating light.

For the case when θ = 70°, the quasi-BIC mode is particularly strong in CL emission, and has a full-width-at-half-maximum (FWHM) of ~28 nm (see Fig. S8 for details). The local field distribution for this quasi-BIC resonance is given in Fig. 3d, showing a ~23-fold local electric field enhancement factor. Figure 3e presents the corresponding multipolar decomposition, showing how electric dipole (ED), magnetic dipole (MD), electric quadrupole (EQ) and magnetic quadrupole (MQ) contributions are excited. The magnetic dipole is directed along the *z*-direction (MD_*z*_), while the electric dipole lies in-plane along the *x*-direction (ED_*x*_), allowing the in- and out-coupling of the incident plane-wave radiation. Therefore, the nano-optical setup of STEM-EELS in combination with CL is able to differentiate and characterize both non-radiative “true” BIC resonances as well as the radiative quasi-BIC resonances.

When the antenna tilt angle θ is changed from 80° to 70°, the *Q* factor will be reduced due to the increased symmetry breaking^19^. This phenomenon is confirmed on the experimentally measured CL emission as shown in Fig. 3c, with a detailed analysis in Figs. S8 and S9. It shows that when the tilt angle θ is changed from 80° to 70°, the *Q* factor is reduced from ~32 to ~26 with the corresponding FWHM being changed from ~23 nm to ~28 nm.

There exists a slight resonance shift between the measured EELS/CL spectra in Fig. 3a, c, with respect to the measured optical reflectance in Fig. 3b. This slight resonance shift originates from imperfections introduced in the nanofabrication process and from the difference in measurement principles. Firstly, the BIC antenna array was fabricated on 30-nm-thick, suspended Si_3_N_4_ membranes that are not perfectly flat due to its ultra-thin nature. During fabrication, this curvature will introduce slight local variations in shapes and dimensions of the fabricated silicon BIC nanoantenna arrays. Secondly, the reflectance spectrum in Fig. 3b is measured from a large array of 130 × 130 elements, making this far-field measurement less sensitive to local variations in antenna size and shape.

**Fig. 4:**
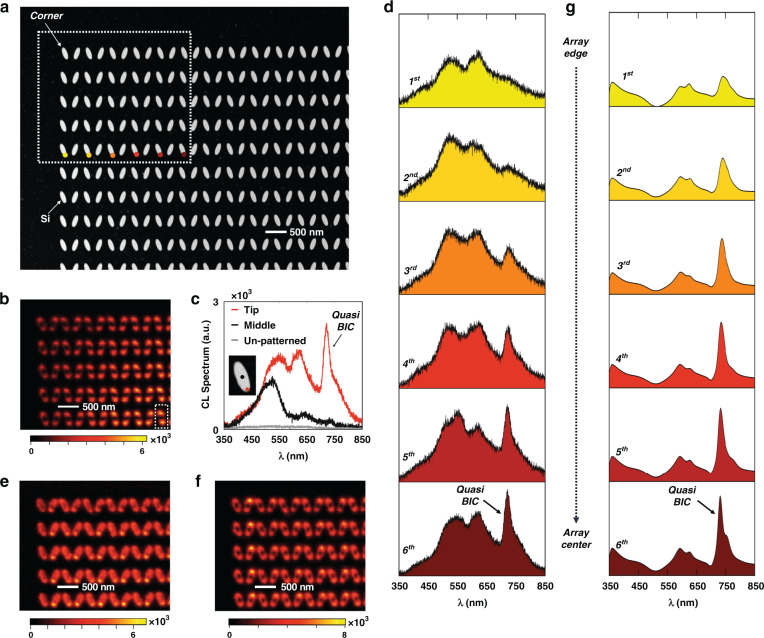
Characterizing the coherent interaction length of the quasi-BIC resonance in a Si nanoantenna array with a tilt angle θ of 70°. **a** STEM HAADF image, showing the corner of a fabricated Si nanoantenna array with 130 × 130 elements. The white dotted rectangle highlights the area used for the CL mapping experiment and the colored dots denote the measurement positions for investigating the CL spectrum evolution. **b** CL intensity map at 722 nm, the quasi-BIC resonance wavelength; note the lower emission closer to the array edge. The wavelength bandwidth for the CL integration is 17 nm. **c** CL spectra from the “tip” position and “middle” position of one selected antenna as highlighted by the white dashed line in (**b**). The 722 nm emission at the “tip” position is strongly enhanced due to the quasi-BIC resonance. The CL spectrum as measured from un-patterned 90-nm-thick Si film is plotted in gray for benchmarking. **d** Evolution of the measured CL emission spectra when going from the array edge towards the array center. It shows that the quasi-BIC resonance requires the presence of neighboring array antennas. **e**, **f** CL maps at the Mie resonance wavelengths of the individual antennas **(e**) 522 nm and (**f**) 614 nm; note the uniform emission all the way to the array edge. **g** Simulations of the CL spectrum evolution

One important feature of the quasi-BIC resonance in nanoantenna arrays is that it is a collective array mode, the formation of which involves neighboring nanoantenna elements. To show this collective effect, we investigate the coherent interaction length of the quasi-BIC resonance based on the CL characterization results in a Si nanoantenna array. No EELS mapping was used here, as the monochromated EELS signal is much weaker and thus requires extremely long data acquisition times for such a large number of antennas. The CL mapping was carried out on a Si nanoantenna array containing 130 × 130 antenna elements, the corner of which is shown in the STEM high-angle annular dark-field (HAADF) image in Fig. 4a, where the Si nanoantenna array has a tilt angle θ of 70°.

Figure 4b was obtained by scanning the electron beam in the white dotted rectangle of Fig. 4a and recording a CL spectrum at each pixel in the scan. This three-dimensional CL data set is then stored and processed as described in the Methods section, resulting in Fig. 4b the spatial distribution of the CL emission at the quasi-BIC resonant wavelength of ~722 nm, using an integration bandwidth of 17 nm. Figure 4c presents the CL spectra measured from a single antenna element indicated in the white dashed rectangle in Fig. 4b. It shows that the CL emission measured at the tip position of the silicon nanoantenna (in red) is significantly different from the CL spectrum measured at the middle position (in black); these respective measurement positions are indicated on the inset STEM image in Fig. 4c. For comparison, the graph also plots the CL emission measured from a 90-nm thick, *unpatterned* Si film; its much lower CL intensity is measured to give a 63 times weaker emissivity as compared to the Si array.

The CL map in Fig. 4b gives direct visual evidence of the coherent interaction length of the quasi-BIC resonance due to the collective antenna array effect. As seen there, the CL emission at the quasi-BIC (~722 nm wavelength) is very weak at the edge of the antenna array, and it becomes stronger when the electron beam moves towards its center. This edge effect becomes even more obvious when we plot the CL spectra as a function of the distance to the edge of the array, shown in Fig. 4d. The 1st antenna element at the array edge shows no sign of the quasi-BIC resonance at all. Our CL experiments demonstrate that the quasi-BIC mode is only fully established after at least six antenna elements into the array; the outer six elements only exhibit a partially established quasi-BIC mode. This observation is in stark contrast with the CL map at the Mie resonances of the nanoantennas shown in Fig. 4e, f. As these resonances are supported by individual antennas, we observe no emission reduction or enhancement as a function of location in the nanoantenna array.

To corroborate this understanding, Fig. S10 presents simulations of the CL emission as enhanced by the quasi-BIC resonance. We adopted the approach from Das et al.^56^, performing FDTD simulations with the incident electron beam modeled as an array of *z*-polarized dipole sources with an appropriate phase delay. Here, to account for the spectral broadening effect due to the limited CL spectral resolution, Fig. 4g was obtained after the convolution with a normalized Gaussian function (see details in “Methods” section). These simulation results in Fig. S10 and Fig. 4g verify the experimental observations in Fig. 4a–d, showing that the first six antenna elements at the edge of the array only have partially established quasi-BIC resonances. In addition, the EELS calculations for nanoparticle arrays of different sizes shown in Fig. S11 indicate that the interaction coherent length for the “true” BIC resonance is similar to the one extracted from CL measurements and calculations. This indicates that by tilting the antenna orientation, the modes acquire a radiative character, but their nature remains very similar to that of their fully dark counterpart with θ = 90°.

## Conclusions

By combining nanoscale electron beam spectroscopy in the STEM, theoretical simulations and multipolar decomposition, we observe “true” photonic BIC on nanostructured Si samples. This localized probing approach provides several distinguishing advantages over conventional optical excitation approaches in the far-field. For instance, the electron beam-induced CL emission is able to spatially map the coherent interaction length of the quasi-BIC resonance, which, in our case, shows that this mode is only partially established in the outer six antenna elements, and only fully formed beyond the sixth element towards the center of the array. When STEM-CL spectroscopy is combined with monochromated STEM-EELS measurements, the “true” BIC resonance is revealed, even in situations when their excitation is impossible using far-field optical irradiation. Our results provide a general methodology to quantitatively probe the mode formation mechanism for BIC with nanometer spatial precision. It will be broadly applicable to quantify other emerging optical resonances such as localized excitation of quantum emitters, and will provide direct and unique insight into their nature.

## Materials and methods

### Nanofabrication of Si nanostructured on membrane

Amorphous Si with a thickness of 90 nm was grown onto a 30-nm-thick Si_3_N_4_ membrane (Agar Scientific S1711), by using plasma-enhanced chemical vapor deposition (PECVD). Before the electron beam exposure, HSQ resist with a concentration of 2% wt., diluted in methyl isobutyl ketone (Dow Corning XR-1541-002), was spin coated onto the cleaned substrate at 5k round-per-minute (rpm), giving a thickness of ~30 nm. The electron beam exposure was carried out with the following conditions: electron acceleration voltage of 100 keV, beam current of 200 pA, and an exposure dose of ~12 mC/cm^2^. The sample was then developed by a NaOH/NaCl salt solution (1% wt./4% wt. in de-ionized water) for 60 s and immersed in de-ionized water for 60 seconds to stop the development. Next, the sample was immediately rinsed by acetone, isopropanol alcohol (IPA) and dried by a continuous flow of nitrogen. Si etching was then carried out by using inductively coupled-plasma (ICP, Oxford Instruments Plasmalab System 100), with Cl_2_ gas chemistry at 6 ^o^C^57^.

### CL and EELS in a STEM

The STEM-CL spectroscopy and STEM-HAADF imaging experiments were carried out with a FEI Titan with the sample at room temperature. The system is equipped with a Schottky emitter and a Wien-type monochromator that was used to disperse and filter the electron beam to a resolution of 65 meV. EELS spectra were acquired with a Tridiem HR detector in spectrum imaging mode, using binned gain averaging^58^. The background signal was subtracted by fitting a high-quality ‘zero-loss peak’ to the measured spectra between 0.5 and 1.0 eV, well before the first onset of the experimental peaks. An example of the EELS background fitting is shown in Fig. S12. A Gatan Vulcan (sample holder and light detection system) was used for CL measurements. The electron beam was accelerated at 200 keV with the monochromator being turned off, while the electron beam was focused into a probe approximately 1 nm in diameter. The CL maps were acquired with a diffraction grating blazed at 500 nm and a dwell time set to 0.42 s per spectrum, where the CL spectral resolution is 17 nm, limited by the slit aperture of the spectrometer. Data processing consisted of subtracting the CCD dark-noise read-out and removing strong single-channel ‘X-ray’ spikes.

### Reflectance simulations and multipolar decomposition

Finite-difference time-domain (FDTD) simulations were carried out using a commercial software (Lumerical FDTD Solutions). In order to consider the random deformation of the ultra-thin Si_3_N_4_ membrane (i.e. 30 nm in thickness) during the ICP CVD process for growing Si film, 13 × 13 arrays of Si nanoantennas were used with the incident optical field being linearly polarized along *x*-direction at normal incidence condition. A non-uniform meshing technique was applied with a minimum mesh size down to 0.5 nm. The dielectric function of amorphous Si was taken from the measured *n* and *k* values by ellipsometry^59^. Moreover, multipolar decomposition analysis was carried out to analyze the optical modes being excited within the structures, where this multipolar decomposition was implemented by Finite Element Method (FEM) in COMSOL. The detailed formulas of multipolar analysis can be found in the supporting information of the reference^59^.

### CL simulations

The simulation of CL emission was based on a finite-difference time-domain (FDTD) approach as reported in the literature^56^, where an array of dipole sources is able to simulate the CL emission process. Fig. S10a presents a schematic, using an array of 41 *z*-polarized dipole sources distributed with a uniform separation of 50 nm along the *z*-direction and having a relative phase-shift following the expression ${\rm{e}}^{{\rm{i}}\omega z/\nu }$. Here, *ν* denotes the electron speed, $\omega$ denotes the oscillation frequency and *z* denotes the dipole position^56^. These 41 *z*-polarized dipole sources are placed symmetrically with respect to the 30-nm-thickness membrane. In our TEM setup, the acceleration electron voltage is 200 kV, and the electrons are traveling at a speed of ~0.695c. To record the CL emission, a monitor is placed at a *z*-plane, which is 500 nm above the top surface of the 13 × 13 elliptic cylinder nanoarray. In order to remove the direct emission of these dipole sources, we take the vectorial subtraction between the total electrical field components with and without the elliptic cylinder nanoarray. After that, a near-to-far transformation was carried out to simulate the collected CL emission within a solid angle of (−15^o^, 15^o^). Detailed CL simulation results are shown in Fig. S10. In addition, to account for the spectral broadening effect due to the limited spectral resolution for CL measurements, these simulated CL spectra were convoluted with a normalized Gaussian function of 16 meV width to get the simulated CL emission profiles as shown in Fig. 4g.

### EELS simulation

The numerical calculations were performed under the finite-element solver of Maxwell’s Equations in frequency domain implemented in COMSOL Multiphysics. The frequency-dependent electron beam propagating along *z*-direction at position (*x*_*e*_, *y*_*e*_) was simulated by a line current of the form $j(r,\omega ) = e\,\exp \left\{ { - \frac{{i\omega z}}{v}} \right\}\delta (x - x_e)\delta \left( {y - y_e} \right)\hat z$, where $e$ is the electron charge and the electron velocity, $v$, was set in accordance with the experiments. The energy loss probability is then calculated as $A = \Gamma = \frac{1}{{\pi \hbar \omega }}{\int} {{\rm{d}}z\,{\it{{\rm{Re}}}}\left\{ {j^ \ast (r,w)E_{{\rm{SC}}}(r,\omega )} \right\}}$, where ***E***_sc_ (***r***, ω) are the electric fields scattered by the dielectric nanoparticle array^40^. The EELS spectra were calculated by convolving ${{\Gamma }}\left( \omega \right)$ with a normalized Gaussian function of 65 meV width, mimicking the experimental spectral resolution. Detailed EELS simulations for different array sizes are shown in Fig. S11 for the case θ = 90°.

## Supplementary information

Supplementary Information

Supporting information Video
