# Supplementary material for: Nanoscale mapping of optically inaccessible bound-states-in-the-continuum
Source: Light Sci Appl. 2022 Jan 20;11:20. doi: 10.1038/s41377-021-00707-2 (PMC8776833; doi:10.1038/s41377-021-00707-2)
Supplement: Supplementary file 1 — Supplementary Information [file 41377_2021_707_MOESM1_ESM.docx]

Supplementary Information for

**Nanoscale Mapping of Optically Inaccessible Bound-States-in-the-Continuum**

Zhaogang Dong^1,2,†,*^, Zackaria Mahfoud^1,†^, Ramón Paniagua-Domínguez^1^, Hongtao Wang^3^, Antonio I. Fernández-Domínguez^4^, Sergey Gorelik^1,5^, Son Tung Ha^1^, Febiana Tjiptoharsono^1^, Arseniy I. Kuznetsov^1^, Michel Bosman^1,2,*^, and Joel K. W. Yang^1,3,*^

^1^Institute of Materials Research and Engineering, A*STAR (Agency for Science, Technology and Research), 2 Fusionopolis Way, #08-03 Innovis, 138634 Singapore

^2^Department of Materials Science and Engineering, National University of Singapore, 9 Engineering Drive 1, 117575, Singapore

^3^Singapore University of Technology and Design, 8 Somapah Road, 487372, Singapore

^4^Departamento de Física Teórica de la Materia Condensada and Condensed Matter Physics Center, Universidad Autónoma de Madrid, 28049 Madrid, Spain

^5^Singapore Institute of Food and Biotechnology Innovation, A*STAR (Agency for Science, Technology and Research), 31 Biopolis Way, #01-02 Nanos, 138669, Singapore

*Correspondence and requests for materials should be addressed to J.K.W.Y. (email: [joel_yang@sutd.edu.sg](mailto:joel_yang@sutd.edu.sg)), M.B. (email: [msemb@nus.edu.sg](mailto:msemb@nus.edu.sg)) and Z.D. (email: [dongz@imre.a-star.edu.sg](mailto:dongz@imre.a-star.edu.sg)).


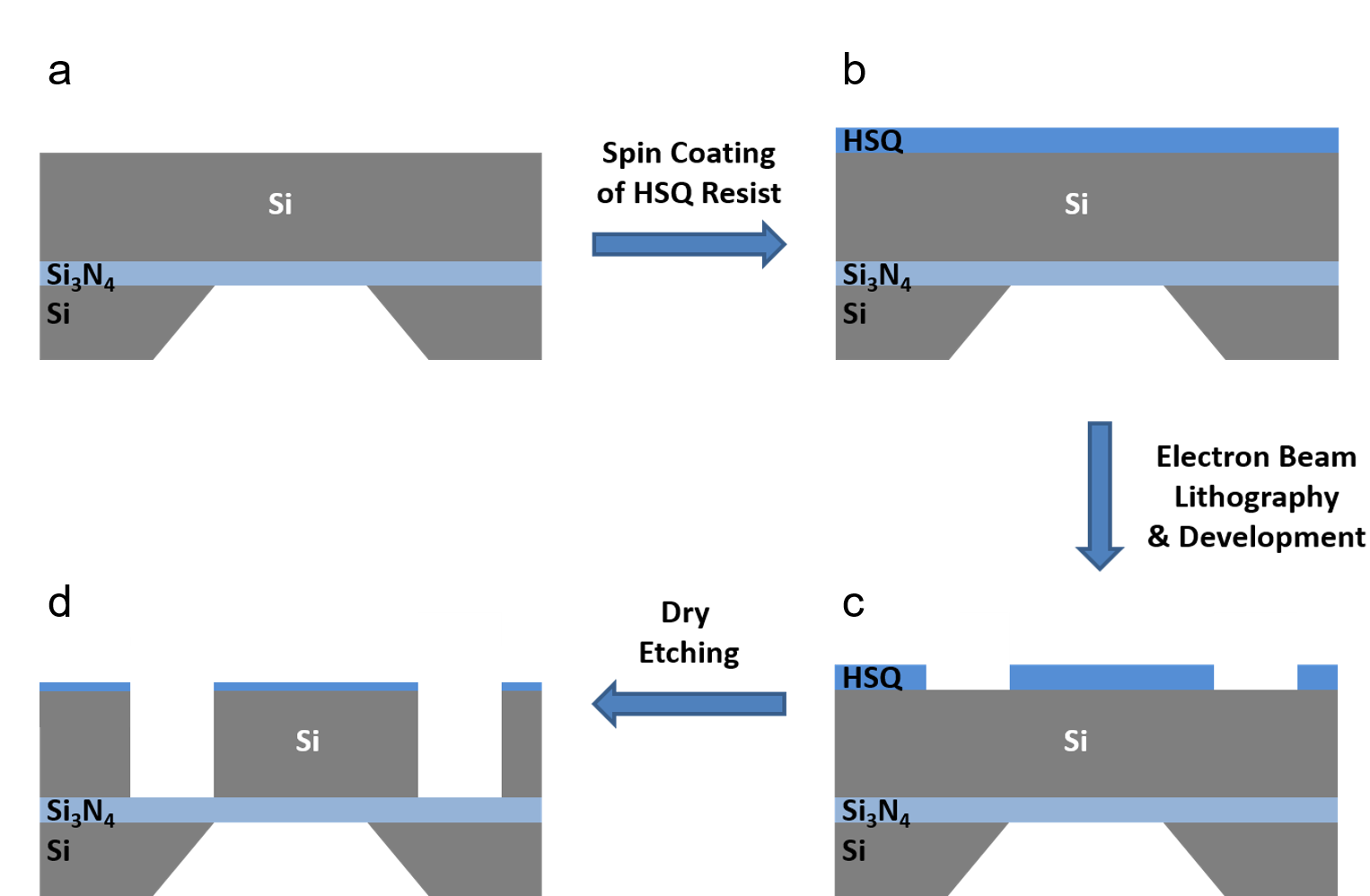


**Fig. S1. Fabrication process of amorphous silicon nanostructures on a 30‑nm‑thick Si_3_N_4_ membrane. a** 90-nm-thick amorphous silicon film is grown onto the 30‑nm‑thick amorphous Si_3_N_4_ membrane by using plasma-enhanced chemical vapor decomposition (PECVD). **b** 30‑nm‑thick hydrogen silsesquioxane (HSQ, 2%, Dow Corning XR‑1541‑002) resist is spin coated onto the amorphous silicon film surface at a speed of 5k rpm. **c** E-beam exposure to fabricate the HSQ resist mask. **d** Inductively-coupled plasma (ICP) for silicon etching with Cl_2_ gas chemistry.

**
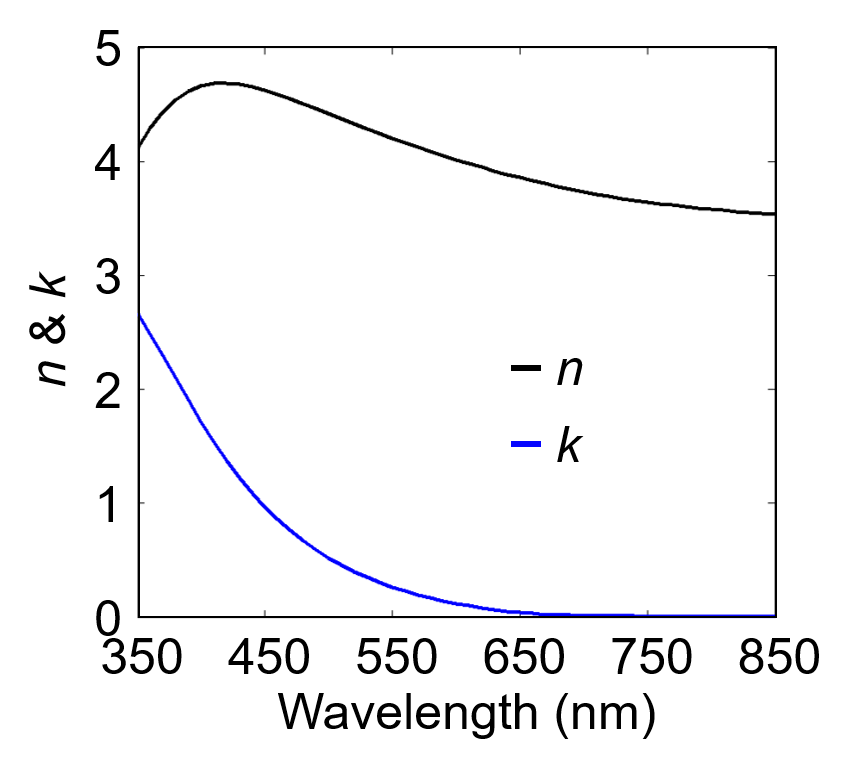
**

**Fig. S2. Measured refractive index (n) and** **extinction coefficient (k) for the amorphous silicon film as grown by using ICP CVD.**


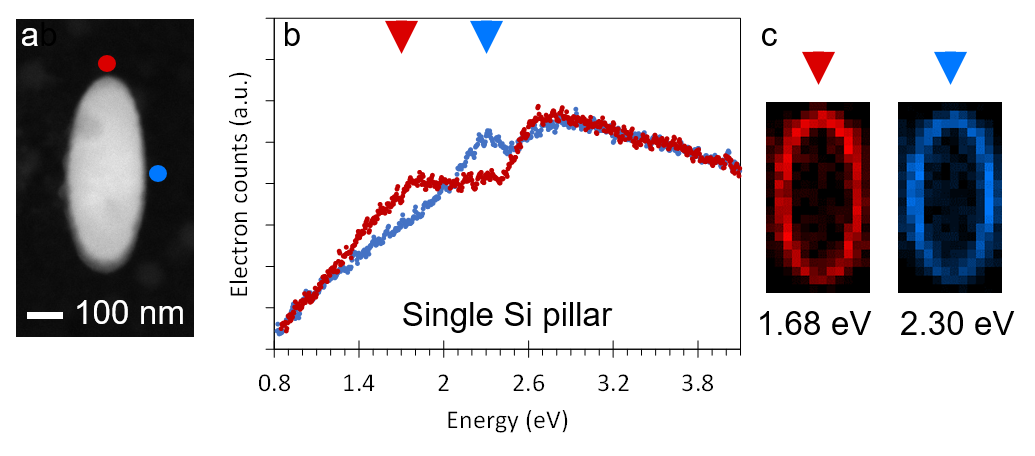


**Fig. S3. Monochromated EELS measurements for a non-array, single nanoantenna.** This single antenna is 3 µm away from any other antennas. **a** STEM image. **b** EELS spectra measured by placing the small electron beam at the tip (red) or at the side (blue) of the antenna. **c** EELS maps at 1.68 (red) and 2.30 eV (blue). If this antenna were in an array, the red map would have shown strong extinction at the tips, similar to that in Fig. S5 for the 90° case. However, for the current case, the red EELS map in Fig. S3c does not show the BIC characteristics with the enhanced extinction at the tips of the antennas. Therefore, no BIC resonance is established for this single antenna.

**
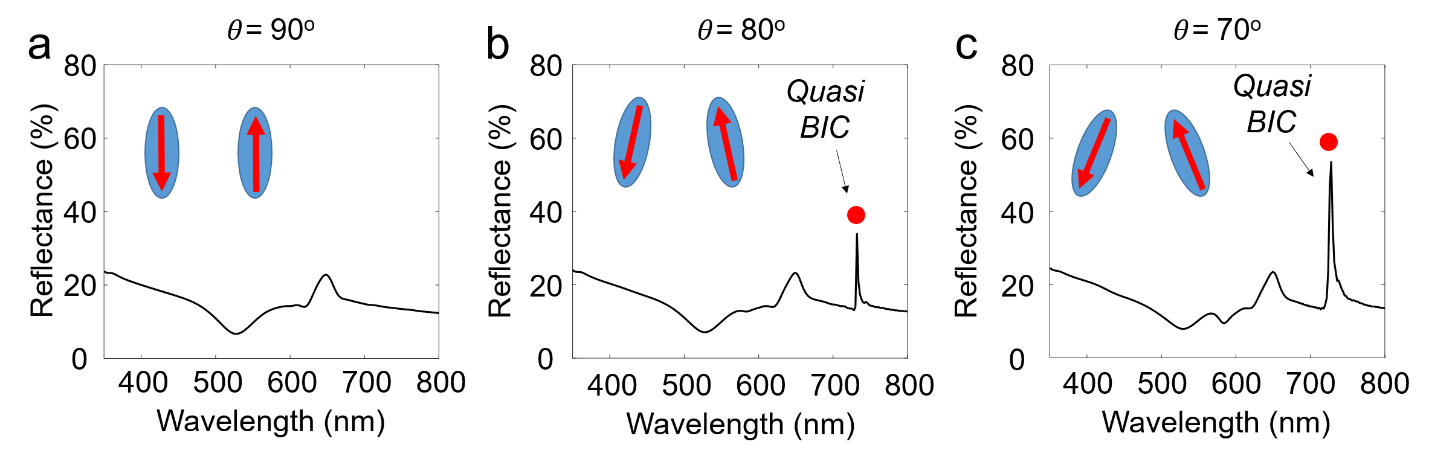
**

**Fig. S4. Simulated reflectance spectra of a pair of** **elliptic cylinder nanoantennas with different tilt angle θ.** The excitation optical field is *x*-polarized. **a** θ=90^o^. **b** θ=80^o^. **c** θ=70^o^. It shows that the “true” BIC resonance cannot be excited when the angle θ is 90^o^. The inset figures schematically present the effective dipole moments induced in the silicon array elements at the “true” BIC and quasi-BIC condition.

**
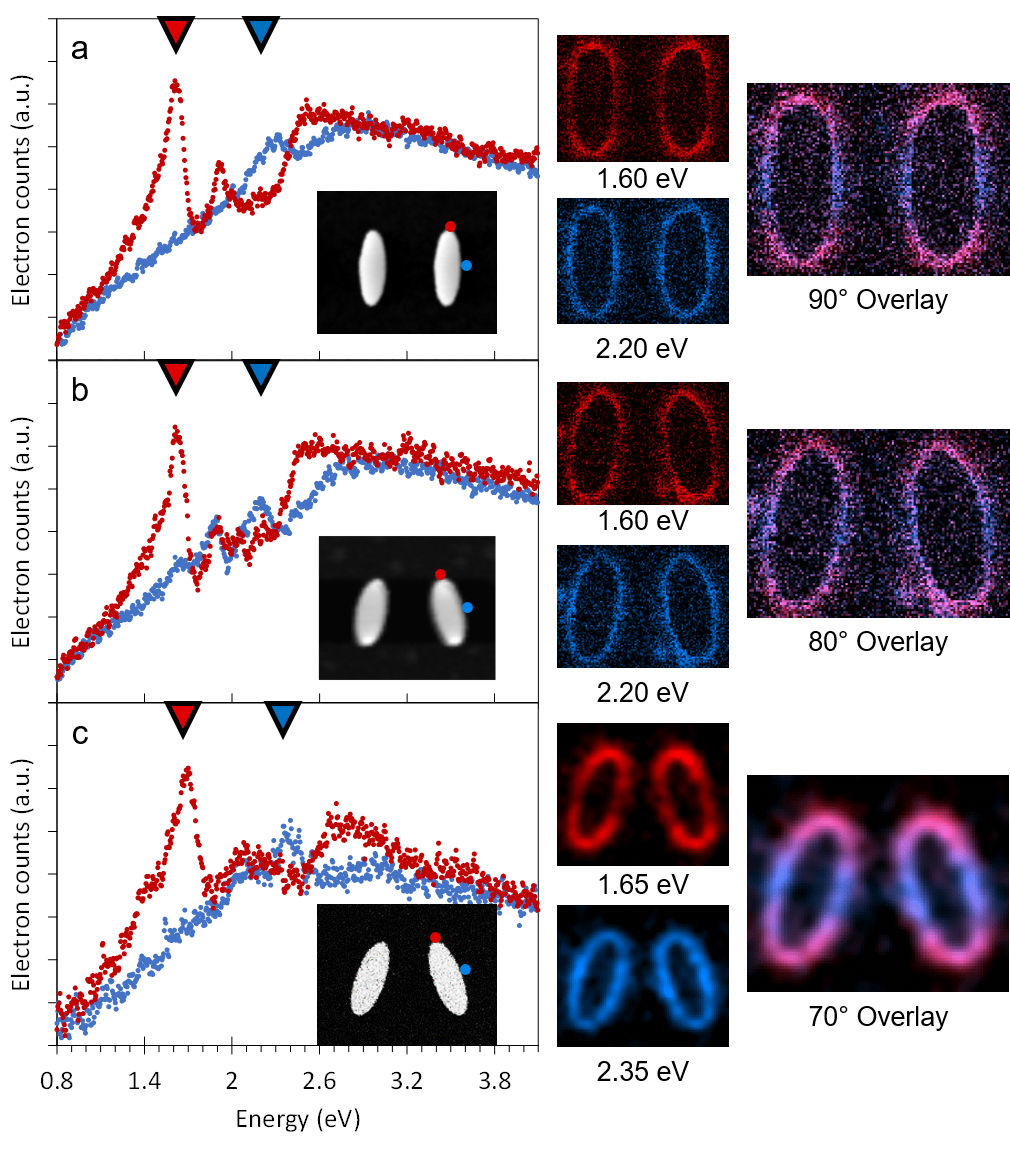
**

**Fig. S5. Monochromated EELS spectra and maps at three different tilt angles. a** θ=90°. **b** θ=80°. **c** θ=70°. The red and blue spectra were taken by placing the ~2 nm electron beam at the tip and side of the antenna respectively, as indicated in the STEM inset. The BIC mode in (a) occurred at 1.60 eV (λ=775 nm), and can be recognized in the maps by its stronger extinction at the antenna tips. In comparison, the EELS map for the Mie resonances in blue do not show this longitudinal directionality. For the θ=70° case at (c), the Mie resonance is at 2.35 eV (λ=528 nm).

**
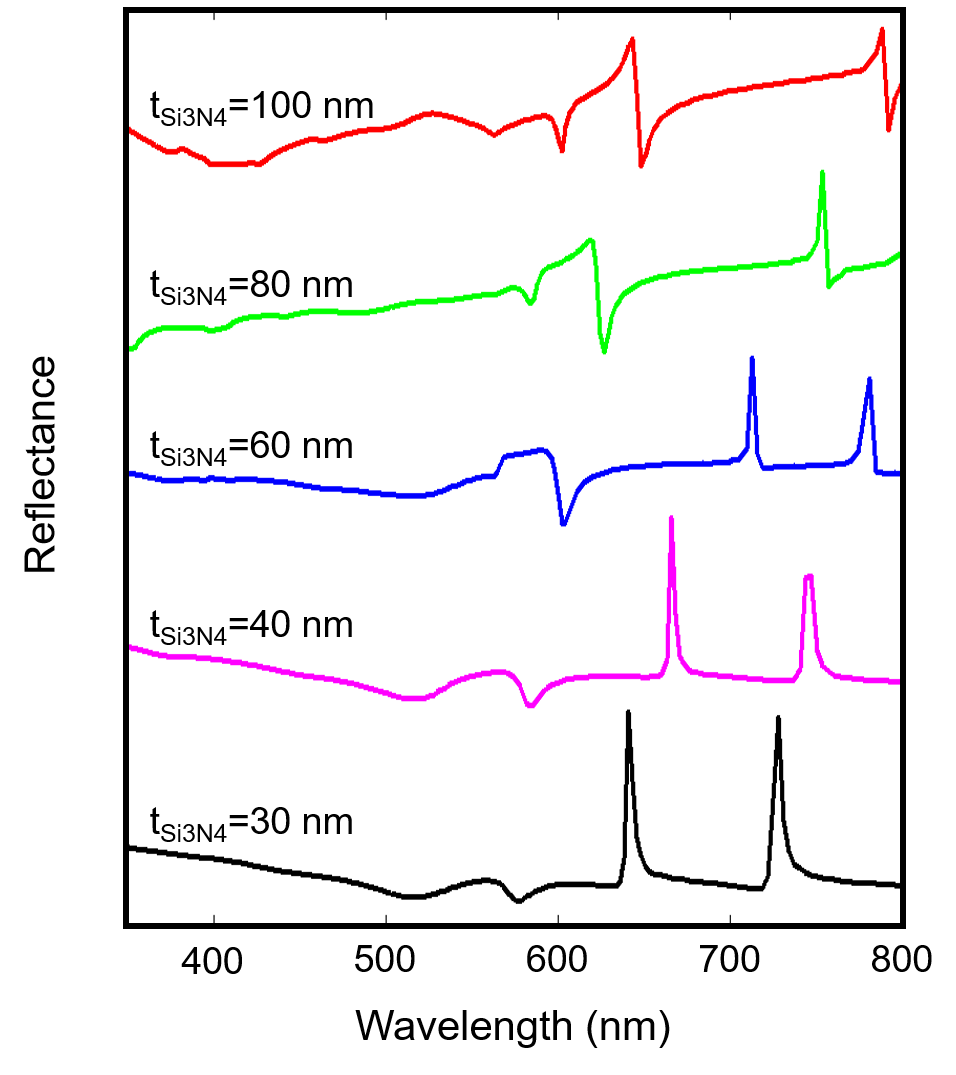
**

**Fig. S6. Simulated reflectance spectra when the thickness of the Si_3_N_4_ membrane is varying from 30 nm to 40 nm, 60 nm, 80 nm and 100 nm.** The increment of membrane thickness will red shift all the optical resonance modes.


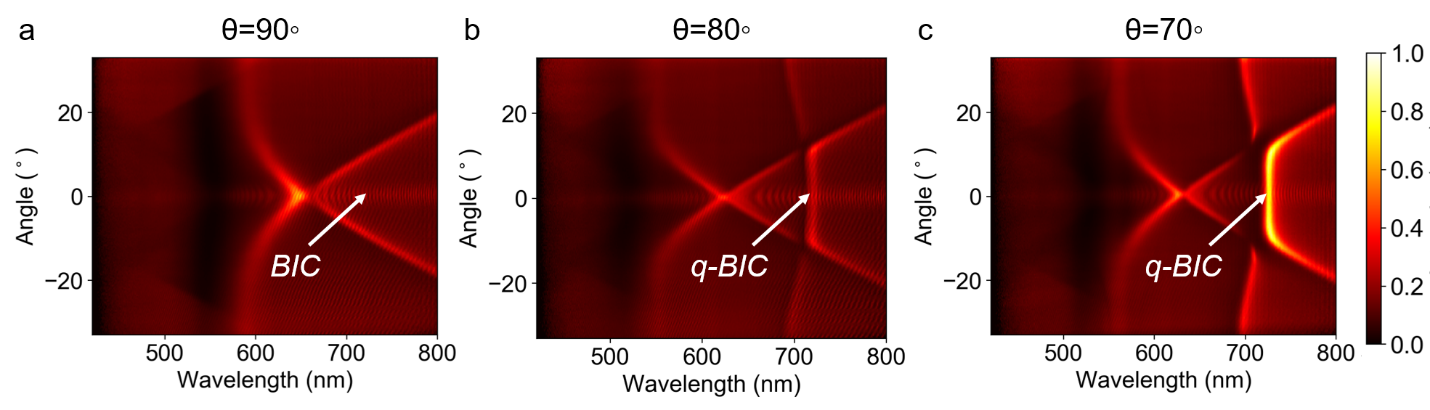


**Fig. S7. Angle-resolved reflectance spectra at the back focal plane under *x*‑polarized condition for elliptic cylinder nanoantennas with the respective tilt angles.** **a** θ=90^o^. **b** θ=80^o^. **c** θ=70^o^. It shows that the true BIC modes are inaccessible by far-field propagating light when the tilt angle θ is 90^o^, as shown in Fig. S3(a).

**Supplementary Note 1. Fitting of CL emission profiles with Lorentz oscillator model.**

For the CL emission profile as shown in Fig. 3(c) for θ=70^o^, it could be fitted with three Lorentzian profiles with the following equations:

$CL=C_{1}\times\frac{\omega_{p}^{2}\gamma_{1}\omega}{\left( \omega_{1}^{2}-\omega^{2} \right)^{2}+\omega^{2}\gamma_{1}^{2}}+C_{2}\times\frac{\omega_{p}^{2}\gamma_{2}\omega}{\left( \omega_{2}^{2}-\omega^{2} \right)^{2}+\omega^{2}\gamma_{2}^{2}}+C_{3}\times\frac{\omega_{p}^{2}\gamma_{3}\omega}{\left( \omega_{3}^{2}-\omega^{2} \right)^{2}+\omega^{2}\gamma_{3}^{2}}$, (S1)

where *ω* represents the frequency. $\omega_{1}$, $\omega_{2}$ and $\omega_{3}$ are the resonant frequencies of the three Lorentz oscillator models. $\gamma_{1}$, $\gamma_{2}$ and $\gamma_{3}$ are to denote the respective damping coefficients, and $\omega_{p}$ represents the plasma frequency of silicon. The fitted values of $\omega_{1}$, $\omega_{2}$ and $\omega_{3}$ are 1.718 eV, 1.987 eV and 2.34 eV. In addition, the fitted values of $\gamma_{1}$, $\gamma_{2}$ and $\gamma_{3}$ are 0.067 eV, 0.23 eV and 0.59 eV. Therefore, the CL emission profile due to the quasi BIC mode at 1.718 eV (*i.e.* ~722 nm) has a full-width-at-half-maximum (FWHM) of ~28 nm.


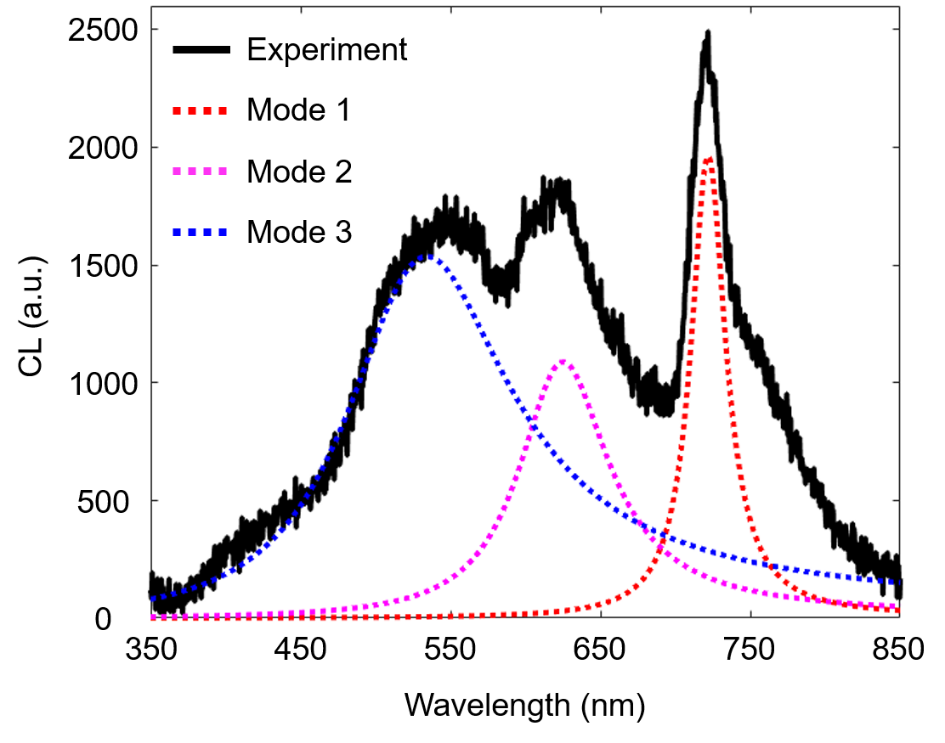


**Fig. S8. Fitting of the measured CL emission from an elliptic cylinder nanoantenna array with tilt angle θ of 70^o^.** The CL emission profile due to the quasi BIC mode at 1.718 eV (*i.e.* ~722 nm) has a full-width-at-half-maximum (FWHM) of ~28 nm, where the corresponding *Q*‑factor is ~26.


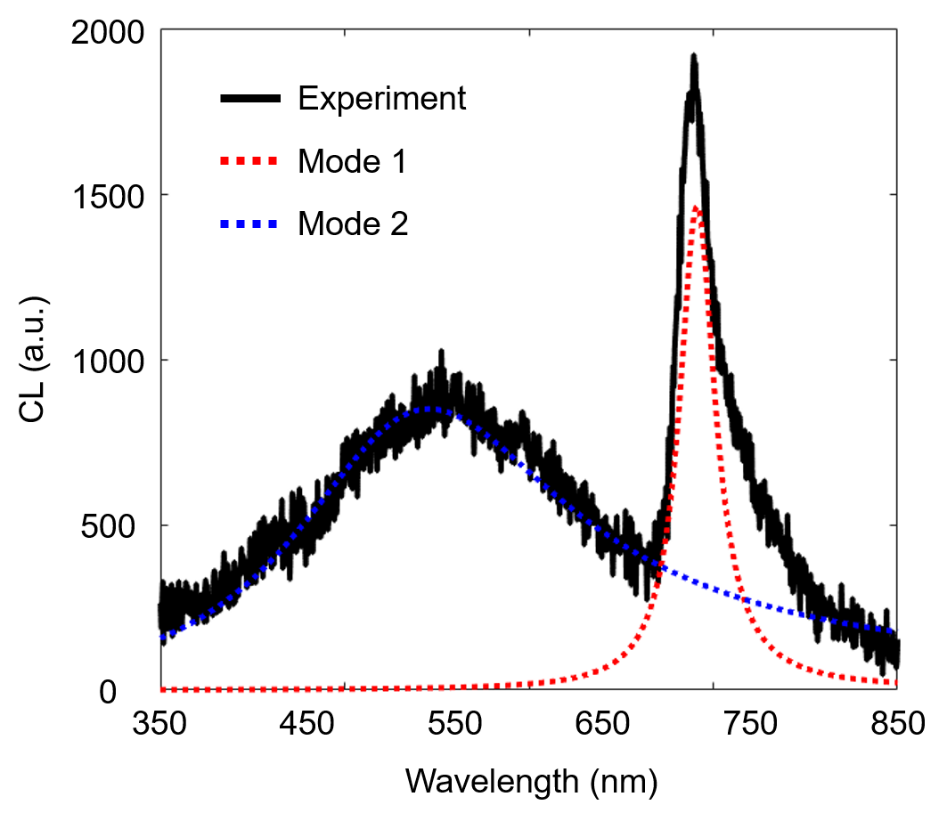


**Fig. S9. Fitting of the measured CL emission from a elliptic cylinder nanoantenna array with tilt angle θ of 80^o^.** The CL emission profile due to the quasi BIC mode at 1.673 eV (*i.e.* ~741 nm) has a full-width-at-half-maximum (FWHM) of ~23 nm, where the corresponding *Q*‑factor is ~32.

**
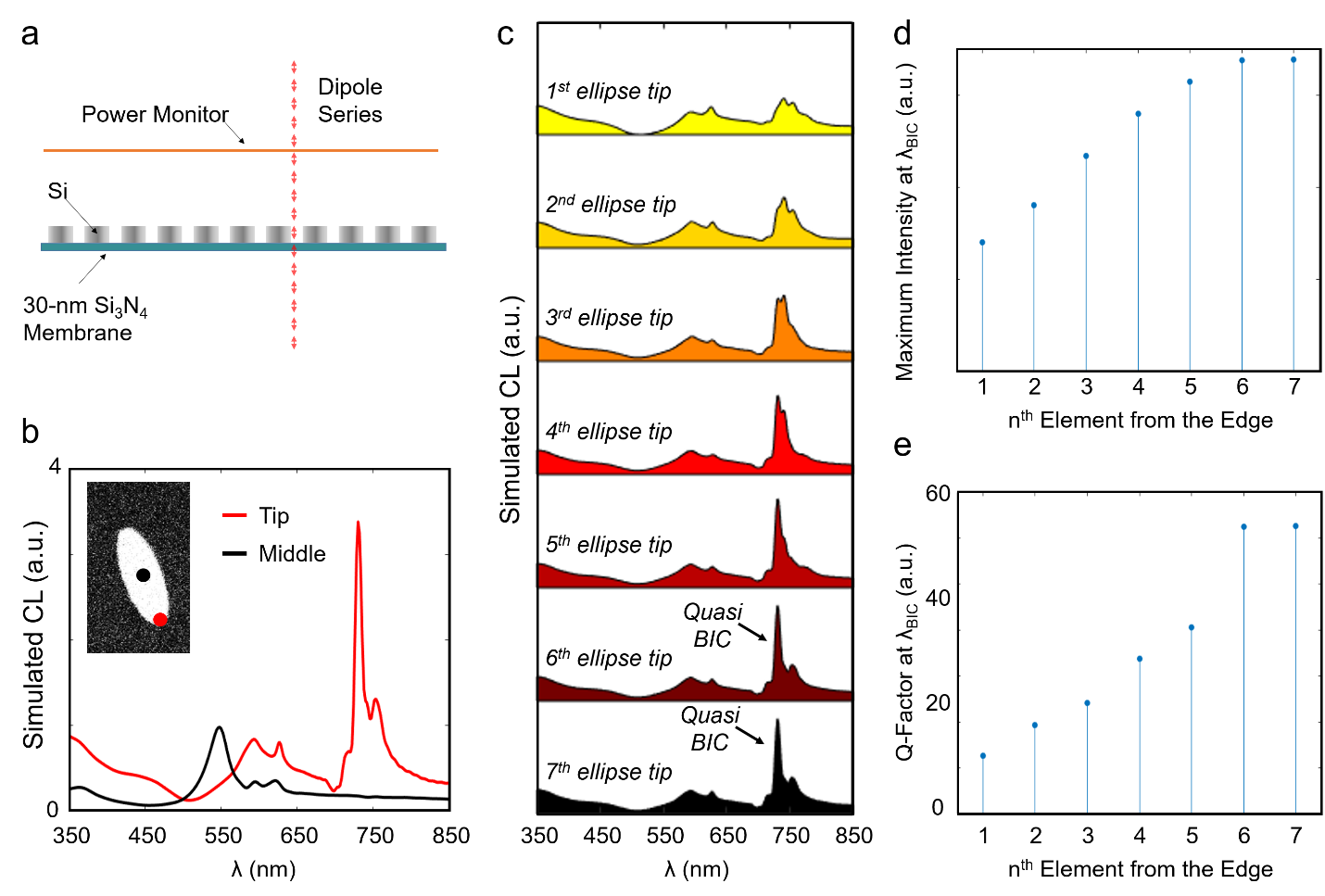
**

**Fig. S10. Simulation of the CL emission spectra with quasi BIC resonance. a** Schematic of the *z*-polarized dipole array for simulating the high energy electron beam that excites the CL emission. **b** Simulated CL spectra when the dipole array is located at the “tip” position and “middle” position of the nanoantennas with a tilt angle θ of 70^o^. The simulated CL emission from the “tip” position has a peak around ~720 nm due to the quasi‑BIC resonance. **c** Simulated CL spectrum evolution at the “tip” position of a nanoantenna, from the array edge to the array center. The array has 13×13 elements. It shows that the quasi-BIC resonance is confined within the center of the array, in agreement with the experimental observation shown in Fig. 4d. **d**-**e** Evolution of the maximum CL intensity and *Q*-factor at λ_BIC_ for the n^th^ antenna element from the edge.

**
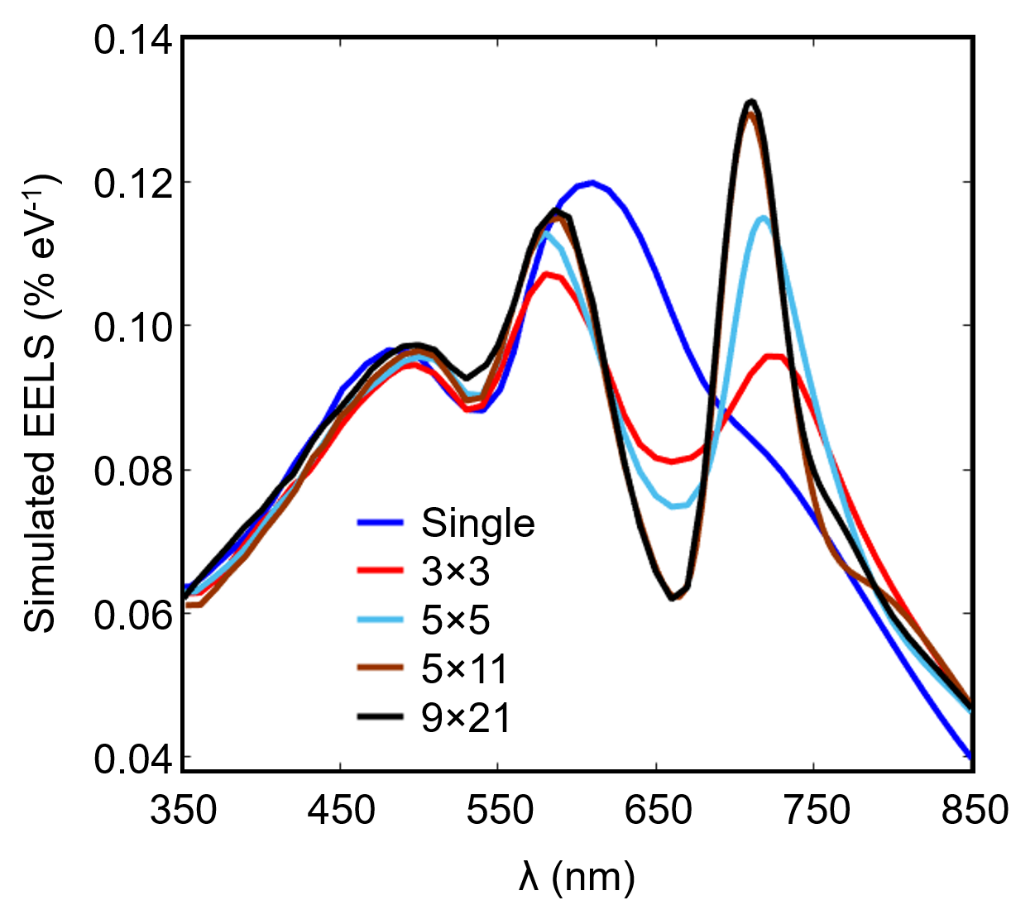
**

**Fig. S11. Simulated EEL spectra from elliptic cylinder nanoantenna arrays of different dimensions with θ=90^o^**. The array dimensions, N×M in the legend indicates the number of elements along the directions defined by the long (N) and short (M) axes of the Si nanoantennas. It shows how the (Quasi) BIC feature in the spectrum builds up as the number of elements in the array increases.

**
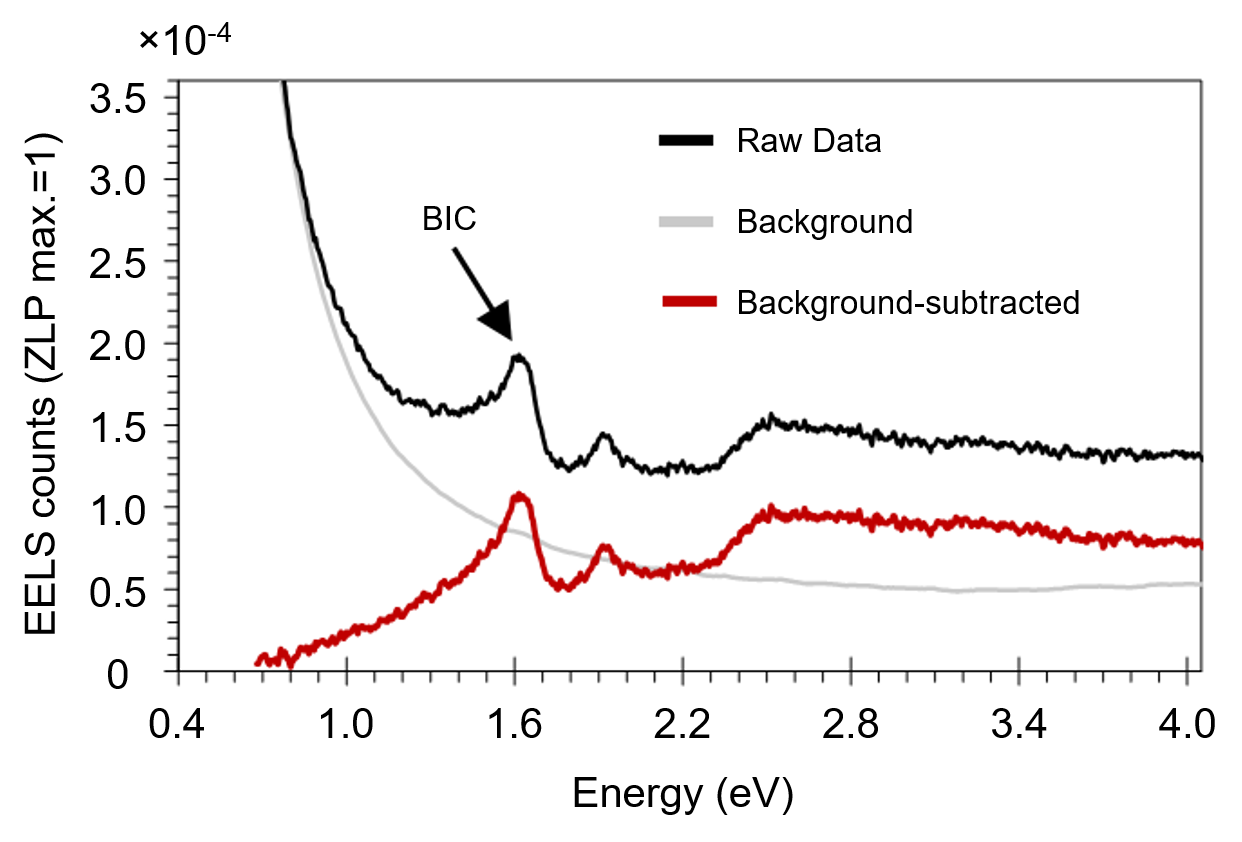
**

**Fig. S12. Background subtraction of ‘zero-loss peak’ in the measured EELS spectrum for the nanoantenna array with θ=90^o^**. The black line presents the raw data of the measured EELS spectrum for the nanoantenna array with θ=90^o^, which includes the background signal due to the tail of the ‘zero-loss peak’. This background signal (grey line) is subtracted by fitting a high-quality zero-loss peak spectrum to the measured spectra between 0.5 and 1.0 eV, well before the first onset of the experimental peaks. After background-subtraction, the remaining signal (red line) is used for the analysis. The high-quality background spectrum was measured from a sample area a few tens of micrometers away from the silicon nanoantennas.

**Supplementary Note 2. Near-field oscillation characteristic of this BIC mode.**

The supplementary video file “NF_BIC.gif” presents the near-field oscillation characteristic of the “true” BIC mode as analyzed in Figure 2. It shows that the neighboring unit cells are oscillating in phase, when *k*=0.
