# Supplementary figures and images for: Nanoscale mapping of optically inaccessible bound-states-in-the-continuum
Source: Light Sci Appl. 2022 Jan 20;11:20. doi: 10.1038/s41377-021-00707-2 (PMC8776833; doi:10.1038/s41377-021-00707-2)

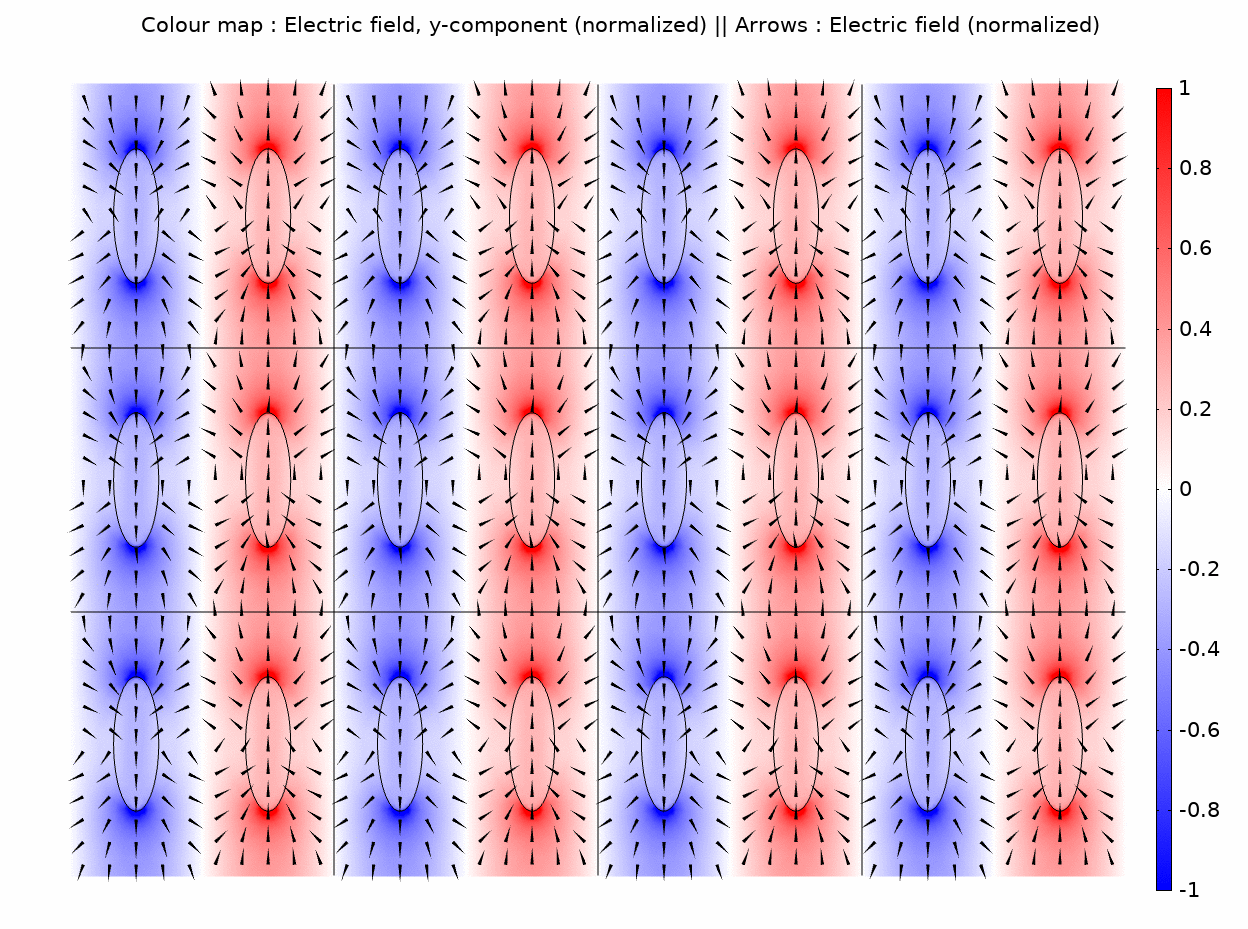

Supplement: Supplementary file 2 — Supporting information Video [file 41377_2021_707_MOESM2_ESM.gif]
